# The cardiopulmonary benefits of physiologically based cord clamping persist for at least 8 hours in lambs with a diaphragmatic hernia

**DOI:** 10.3389/fped.2024.1451497

**Published:** 2024-10-11

**Authors:** Paige J. Riddington, Philip L. J. DeKoninck, Marta Thio, Calum T. Roberts, Risha Bhatia, Janneke Dekker, Aidan J. Kashyap, Benjamin J. Amberg, Karyn A. Rodgers, Alison M. Thiel, Ilias Nitsos, Valerie A. Zahra, Ryan J. Hodges, Stuart B. Hooper, Kelly J. Crossley

**Affiliations:** ^1^ The Ritchie Centre, Hudson Institute of Medical Research, Clayton, VIC, Australia; ^2^ Department of Obstetrics and Gynaecology, Monash University, Clayton, VIC, Australia; ^3^ Department of Obstetrics and Gynaecology, Division of Obstetrics and Fetal Medicine, Erasmus MC University Medical Center—Sophia Children’s Hospital, Rotterdam, Netherlands; ^4^ Newborn Research Centre, The Royal Women’s Hospital, Parkville, VIC, Australia; ^5^ Department of Obstetrics and Gynaecology, The University of Melbourne, Melbourne, VIC, Australia; ^6^ Centre for Research Excellence in Newborn Medicine, The Murdoch Children’s Research Institute, Melbourne, VIC, Australia; ^7^ Monash Newborn, Monash Children’s Hospital, Clayton, VIC, Australia; ^8^ Department of Paediatrics, Monash University, Clayton, VIC, Australia; ^9^ Department of Paediatrics, Division of Neonatology, Willem-Alexander Children's Hospital, Leiden University Medical Centre, Leiden, Netherlands

**Keywords:** congenital diaphragmatic hernia, lung hypoplasia, neonatal transition, pulmonary hypertension, pulmonary blood flow, pulmonary vascular resistance

## Abstract

**Introduction:**

Infants with congenital diaphragmatic hernia can suffer severe respiratory insufficiency and pulmonary hypertension after birth. Aerating the lungs before removing placental support (physiologically based cord clamping, PBCC) increases pulmonary blood flow (PBF) and reduces pulmonary vascular resistance (PVR) in lambs with a diaphragmatic hernia (DH). We hypothesized that these benefits of PBCC persist for at least 8 h after birth.

**Methods:**

At ∼138 days of gestation age (dGA), 21 lambs with a surgically induced left-sided DH (∼86 dGA) were delivered via cesarean section. The umbilical cord was clamped either before ventilation onset (immediate cord clamping, ICC, *n* = 9) or after achieving a tidal volume of 4 ml/kg, with a maximum delay of 10 min (PBCC, *n* = 12). The lambs were ventilated for 8 h, initially with conventional mechanical ventilation, but were switched to high-frequency oscillatory ventilation after 30 min if required. Ventilatory parameters, cardiopulmonary physiology, and arterial blood gases were measured throughout the study.

**Results:**

PBF increased after ventilation onset in both groups and was higher in the PBCC DH lambs than the ICC DH lambs at 8 h (5.2 ± 1.2 vs. 1.9 ± 0.3 ml/min/g; *p* < 0.05). Measured over the entire 8-h ventilation period, PBF was significantly greater (*p* = 0.003) and PVR was significantly lower (*p* = 0.0002) in the PBCC DH lambs compared to the ICC DH lambs. A high incidence of pneumothoraces in both the PBCC (58%) and ICC (55%) lambs contributed to a reduced sample size at 8 h (ICC *n* = 4 and PBCC *n* = 4).

**Conclusion:**

Compared with ICC, PBCC increased PBF and reduced PVR in DH lambs and the effects were sustained for at least 8 h after ventilation onset.

## 1 Introduction

Congenital diaphragmatic hernia (CDH) is a congenital defect that occurs during embryonic development and has an incidence of approximately 1/3,000 live births (1). CDH is characterized by a failure of the diaphragm to close, allowing the abdominal organs to herniate into the thoracic cavity which disrupts the growth and development of fetal lungs, resulting in small hypoplastic lungs (2, 3). Structurally, these lungs have less airway branching, thickened alveolar walls, and a reduced surface area for gas exchange (4, 5). The lungs have fewer pulmonary arteries with a reduced total arterial volume and cross-sectional area of the pulmonary vascular bed (6). As a result, CDH infants experience a challenging transition to newborn life. Their poorly compliant and hypoplastic lungs struggle to aerate and provide adequate gas exchange and the pulmonary vascular bed is unable to dilate and accommodate 100% of the right ventricular output. Thus, CDH infants commonly suffer from respiratory insufficiency and persistent pulmonary hypertension of the newborn (PPHN) after birth (7), with PPHN developing in 70% of CDH infants (8), leading to increased mortality and the need for extracorporeal life support (9–11).

In infants diagnosed with a CDH antenatally, clinical guidelines currently recommend intubation at birth to provide rapid respiratory support and to prevent inflation of the stomach that may be located in the chest (12–14). To facilitate routine intubation, the umbilical cord is often clamped immediately after birth to transfer the infant to a resuscitaire for cardiopulmonary support. However, studies in both preterm lambs (15, 16) and lambs with a diaphragmatic hernia (DH) (17, 18) have demonstrated that immediate umbilical cord clamping exposes the newborn to a transient but significant reduction in cardiac output and a period of hypoxia that can be largely avoided by aerating the lung before cord clamping.

Before birth, umbilical venous return provides the majority of the preload for the left ventricle as fetal pulmonary vascular resistance (PVR) is high and pulmonary blood flow (PBF) is low (19). However, as pulmonary venous return becomes the sole source of preload for the left ventricle after birth, if the umbilical cord is clamped before the lungs have aerated, and PBF has increased, the sudden loss of umbilical venous return causes a 30%–50% decrease in cardiac output (15, 20). The cardiac output then remains low until the lungs aerate and PVR decreases enough to sufficiently increase PBF and restore venous return and preload for the left ventricle (15). Logically, this problem of a low cardiac output at birth is considerably greater when lung aeration at birth is delayed and/or if the decrease in PVR and increase in PBF is blunted by a poorly developed pulmonary vascular bed. As a result, immediate cord clamping (ICC) in CDH infants is likely to result in an extended period of reduced cardiac output and poor oxygenation due to a delay in lung aeration (17).

Aerating the lung to reduce PVR and increase PBF before umbilical cord clamping allows PBF (and pulmonary venous return) to immediately replace umbilical venous return as the primary source of preload to the left ventricle, safeguarding against a reduction in cardiac output at birth. This safeguard is known as physiologically based cord clamping (PBCC) and allows the lungs to aerate and PBF to increase gradually, avoiding the low cardiac output and hypoxia associated with ICC (18). We have previously shown that the increase in PBF and reduction in PVR was markedly improved following PBCC in DH lambs and these differences were sustained for at least 2 h after birth (18). This sustained effect of PBCC on PVR was surprising (18) and suggests that ICC may increase the risk of a gradual onset of PPHN; however, it is currently unclear why ICC may increase this risk.

Our primary aim was to determine whether the larger increase in PBF and decrease in PVR in DH lambs exposed to PBCC is sustained for at least 8 h following ventilation onset. We hypothesized that DH lambs exposed to PBCC would have a persistent increase in PBF and reduction in PVR for up to 8 h after birth compared to DH lambs delivered via ICC.

## 2 Materials and methods

### 2.1 Ethical approval

This experiment was performed in accordance with the National Health and Medical Research Council of Australia guidelines for the care and use of experimental animals and was approved by the Monash Medical Centre Animal Ethics Committee. Methodological reporting is provided as per the relevant ARRIVE guidelines (21).

### 2.2 General surgical methods

All the surgical procedures were performed under general anesthesia using an intravenous injection of 20 mg/kg sodium thiopentone (Pentothal, 1 g in 20 ml: Jurox, NSW, Australia) for induction and inhaled isoflurane (1.5%–2.5% in room air/oxygen: Isoflow, Abbot Pty. Ltd., Australia) for maintenance. Pregnant Merino X Border-Leicester ewes (*n* = 14) were intubated and monitored (expired CO_2_, respiratory rate, heart rate, SpO_2_, absence of a corneal reflex) to ensure adequate anesthesia and maternal wellbeing. Following surgery, ewes received 3 days of post-operative analgesia (transdermal fentanyl patches, 75 µg/h: Janssen-Cliag, North Ryde, NSW, Australia) and were monitored daily until delivery.

### 2.3 Diaphragmatic hernia creation

A DH was surgically created in twin fetal lambs (*n* = 28) at ∼86 days of gestational age (dGA; term = 147 dGA) as previously described (17). Briefly, a midline laparotomy was used to expose the uterus and a hysterotomy was performed to exteriorize the fetal head and forelimbs. The fetal chest was incised through the 10th intercostal space to expose and incise the fetal diaphragm, enabling a portion of the stomach and bowels to be positioned into the fetal chest cavity.

### 2.4 Fetal instrumentation and delivery

At ∼138 dGA (near-term), ewes and their fetuses were anesthetized, and the lambs were instrumented prior to cesarean delivery, as previously described (17). Briefly, the lambs were partially delivered to exteriorize the fetal head, chest, and forelimbs and were intubated with a clamped endotracheal tube (ID 4.5 mm; Portex, Ltd., Kent, England). Polyvinyl catheters (ID 0.86 mm, Dural Plastics Inc., Australia) were inserted into the left carotid artery and the main pulmonary artery to position catheter tips in the brachiocephalic artery and the left pulmonary artery, respectively, to continuously record arterial blood pressures. A catheter inserted into the left jugular vein was used for the administration of antibiotics (1,000 mg cefazolin) during surgery and anesthetic and glucose during ventilation. Ultrasonic flow probes (Transonic Systems, Ithaca, NY, USA) were placed around the left carotid artery (size 3 mm) and the left pulmonary artery (size 4 mm) to continuously record blood flows. A near-infrared spectroscopy sensor (NIRS; Foresight, CAS Medical Systems Inc., CT, USA) was placed on the forehead to measure cerebral tissue oxygen saturation. The stomach was drained with a nasogastric tube (8 Fr). After instrumentation, the lambs were delivered from the uterus, dried, and carefully placed on the maternal abdomen to avoid occluding the umbilical cord blood flow. The endotracheal tube was unclamped, lung liquid was passively drained, and the volume of lung liquid was recorded.

Each DH lamb was randomly allocated to either ICC as the control group or PBCC as the experimental group. The umbilical cord was clamped either ∼30 to 60 s before ventilation onset (ICC) or after achieving a tidal volume of 4 ml/kg, with a maximum delay of 10 min (PBCC).

### 2.5 Ventilation

Ventilation commenced with a 30 s sustained inflation (SI, 35 cmH_2_O, 21% O_2_) to promote aeration of the least compliant lung regions, followed by 30 s of intermittent positive pressure ventilation (iPPV) in volume guarantee mode using a target tidal volume (V_T_) of 4 ml/kg (Babylog 8000+; Drager, Luebeck, Germany). The peak inspiratory pressure (PIP) limit was 35 cmH_2_O, and the positive end-expiratory pressure (PEEP) was set at 5 cmH_2_O. If the target V_T_ was not achieved after 30 s of iPPV, a second 30 s sustained inflation was delivered followed by iPPV for a minimum of 30 min. After the umbilical cord was clamped, the lambs were transferred to a neonatal resuscitaire (CosyCot, Fisher & Paykel Healthcare, Panmure, Auckland, New Zealand) and sedated with alfaxalone diluted in 5% glucose (5–15 ml/kg body weight/h; Alfaxan, Jurox New Zealand Pty. Ltd., New Zealand).

Ventilation parameters were adjusted and the fraction of inspired oxygen (FiO_2_) levels were titrated to achieve the following blood gas targets to allow for gentle ventilation for the 8-h experimental period: partial pressure of oxygen (PaO_2_) >40 mmHg, partial pressure of carbon dioxide (PaCO_2_) 60–80 mmHg, and arterial oxygen saturation (SaO_2_) of 80%–95%. After 30 min of iPPV, if the PaCO_2_ level increased beyond 80 mmHg or was >65 mmHg with a pH <7.25 while on maximal ventilation settings, the ventilation was switched to high-frequency oscillatory ventilation (HFOV; Sensormedics 3100A, Vyaire Medical, Inc., Mettawa, IL, USA) with initial settings of 8–10 Hz and the same mean airway pressure as measured on the iPPV. Ventilation parameters were then continuously adjusted to maintain the blood gas targets.

### 2.6 Data analysis

#### 2.6.1 Physiological data collection

Pulmonary and carotid arterial blood flows and pressures and cerebral tissue oxygen saturation (SctO_2_) were continuously recorded using LabChart (ADInstruments, NSW, Australia) and analyzed offline. Values for PBF, PVR, and end-diastolic PBF were corrected for left lung weight when the flow in the left pulmonary artery was measured.

Ventilation onset was designated as “time zero” for both the ICC and PBCC DH lambs. Physiological parameters were averaged across a 20-s epoch collected at set timepoints. A 20-s epoch was taken before the lung liquid was drained for a fetal measurement. Furthermore, 20-s epochs were taken every minute for 20 min immediately after the onset of ventilation and every 10 min thereafter.

Arterial blood gas samples were collected every 5 min from the onset of ventilation for the first 30 min, every 10 min until 2 h, and every 30 min thereafter. PVR and alveolar-arterial difference in oxygen (AaDO_2_) were calculated using the equations shown in Table 1.

**Table 1:** Calculations for derived measures.

Measure	Calculation
PVR	$\frac{\left(\mathrm{PAP}-\mathrm{LAP}\right)}{\mathrm{PBF}}$
AaDO_2_	$\left[\mathrm{Fi}O_{2\;}\times (P_{\mathrm{atm}}-\;P_{H_{2}O})-\;\frac{\mathrm{PaC}O_{2}}{\mathrm{RQ}}\right]-\mathrm{Pa}O_{2}$

PVR, pulmonary vascular resistance; PAP, pulmonary artery pressure (mmHg); LAP, left atrial pressure, assumed to equal 9 mmHg based on previous studies (22); PBF, pulmonary blood flow (ml/min); AaDO_2_, alveolar-arterial difference in oxygen; FiO_2_, fraction of inspired oxygen (%); P_atm_, atmospheric pressure (760 mmHg); P_H2O_, water vapor pressure at body temperature (47 mmHg at 39°C); PaCO_2_, partial pressure of carbon dioxide (mmHg); RQ, respiratory quotient (0.8); PaO_2_, partial pressure of oxygen (mmHg).

#### 2.6.2 Postmortem analysis

The lambs were euthanized (sodium pentobarbitone >100 mg/kg IV; Lethabarb, Virbac Pty. Ltd., Peakhurst, Australia) after completing the 8-h ventilation period or earlier due to ethical end-points (i.e., treatment-resistant pneumothorax). Lambs that developed a pneumothorax received needle aspiration to remove the air within the intra-pleural space. If aspiration did not relieve the symptoms of the tension pneumothorax, the pneumothorax was deemed “treatment resistant.” Postmortem examination determined the presence of a diaphragmatic defect and the degree of herniation of the visceral organs. The body weight of the lambs was recorded, and wet lung weights were expressed as a lung-to-body weight ratio (LBWR).

#### 2.6.3 Statistical analysis

A power analysis was used to determine the experimental group sizes, using PBF as the primary outcome, and was based on previous physiological studies of DH lambs (18). However, as it was likely that fewer DH lambs would survive to 8 h (compared to 2 h), we had to estimate the extra number of lambs required. All statistical analyses and graphical presentations were performed with Prism v10.2.1 (GraphPad Software, San Diego, CA, USA). Normality was determined with the Shapiro–Wilk test and transformations were applied when appropriate. Data are expressed as means ± SEM or median (IQR) for non-parametric data. Fetal baseline characteristics were compared with an independent *t*-test or the Mann–Whitney *U*-test for parametric and non-parametric data, respectively. The female:male ratios were analyzed with Fisher's exact test. Survival curves were analyzed using the Log-rank (Mantel–Cox) test. Mixed-effects analysis using the Holm–Sidak multiple comparisons test compared the effect of group (ICC vs. PBCC), time, and interaction. Area under the curve (AUC; baseline = 0 and fetal values excluded) analysis and subsequent *t*-tests were performed to assess the overall impact of ICC vs. PBCC. AUC bar graphs are presented as an insert in each figure where a significant difference was observed. A *p*-value <0.05 was considered statistically significant.

## 3 Results

### 3.1 Animal surgery

In total, 24 (of 28) fetuses survived to delivery. Of the 24 fetuses, *n* = 9 ICC DH lambs and *n* = 12 PBCC lambs were used for this study. Three DH lambs died during instrumentation surgery.

### 3.2 Baseline characteristics

There were no differences in body weight, lung-to-body weight ratio, or sex (ratio of female to male) between the ICC and PBCC DH lambs. Similarly, there were no differences in fetal PBF or PVR measured prior to delivery. All other fetal cardiopulmonary physiology and arterial blood gas variables were similar between the groups and are summarized in Table 2. The umbilical cord was clamped at 42 ± 12 s before ventilation onset in the ICC DH lambs and 7.8 ± 0.8 min after ventilation onset in the PBCC DH lambs.

**Table 2:** Baseline characteristics, fetal cardiopulmonary physiology, and fetal arterial blood gas status in the ICC and PBCC lambs.

	ICC (*n* = 9)	PBCC (*n* = 12)	*p*-value
Body weight (kg)	4.2 ± 0.1	4.5 ± 0.1	0.114
Female:Male	4:5	7:5	0.670
LBWR	0.012 (0.01–0.015)	0.011 (0.01–0.017)	0.862
PBF (ml/min/g)	0.18 (0.08–0.46)	0.35 (0.16–1.90)	0.175
PVR (mmHg·min/mL/g)	285 ± 90	118 ± 34	0.069
PAP (mmHg)	50.2 (34.8–53.8)	47.1 (43.4–48.6)	0.427
CAP (mmHg)	47.5 ± 2.8	46.9 ± 1.32	0.841
CBF (ml/min/kg)	15.3 (11.4–18.5)	14.0 (11.4–19.4)	0.970
Heart rate (bpm)	141.8 ± 12.43	148.1 ± 7.08	0.640
SctO_2_ (%)	58.3 ± 1.94	58.9 ± 2.5	0.854
pH	7.29 ± 0.007	7.28 ± 0.01	0.706
PaCO_2_ (mmHg)	57.0 ± 2.99	53.6 ± 3.94	0.523
PaO_2_ (mmHg)	21.0 ± 1.48	24.6 ± 1.78	0.158
SaO_2_ (%)	57.7 ± 5.12	65.6 ± 3.53	0.207
Hb (g/dl)	12.52 ± 0.68	12.15 ± 0.54	0.668
Hct (%)	38.4 ± 2.10	35.8 ± 2.17	0.418

ICC, immediate cord clamping; PBCC, physiologically based cord clamping; LBWR, lung-to-body weight ratio; PBF, pulmonary blood flow; PVR, pulmonary vascular resistance; PAP, pulmonary artery pressure; CAP, carotid artery pressure; CBF, carotid blood flow; SctO_2_, cerebral tissue oxygen saturation; PaCO_2_, partial pressure of carbon dioxide; PaO_2_, partial pressure of oxygen; SaO_2_, oxygen saturation; Hb, hemoglobin; Hct, hematocrit.

Data are expressed as mean ± SEM or median (IQR). Fetal baseline characteristics were compared with an independent *t*-test or the Mann–Whitney *U*-test for parametric and non-parametric data, respectively. Sex was analyzed as a categorical variable using Fisher's exact test.

### 3.3 Ventilation protocol

Of the lambs that received only one SI, 100% achieved the target V_T_ while 62% of the lambs that received two SIs achieved the target V_T_. The PBCC DH lambs achieved the target V_T_ at 6 min after ventilation onset (3–12 min), whereas the ICC DH lambs achieved the target V_T_ in 8.5 min (4.5–16.3 min; *p* = 0.5) after ventilation onset.

### 3.4 Survival curves

**Figure 1:**
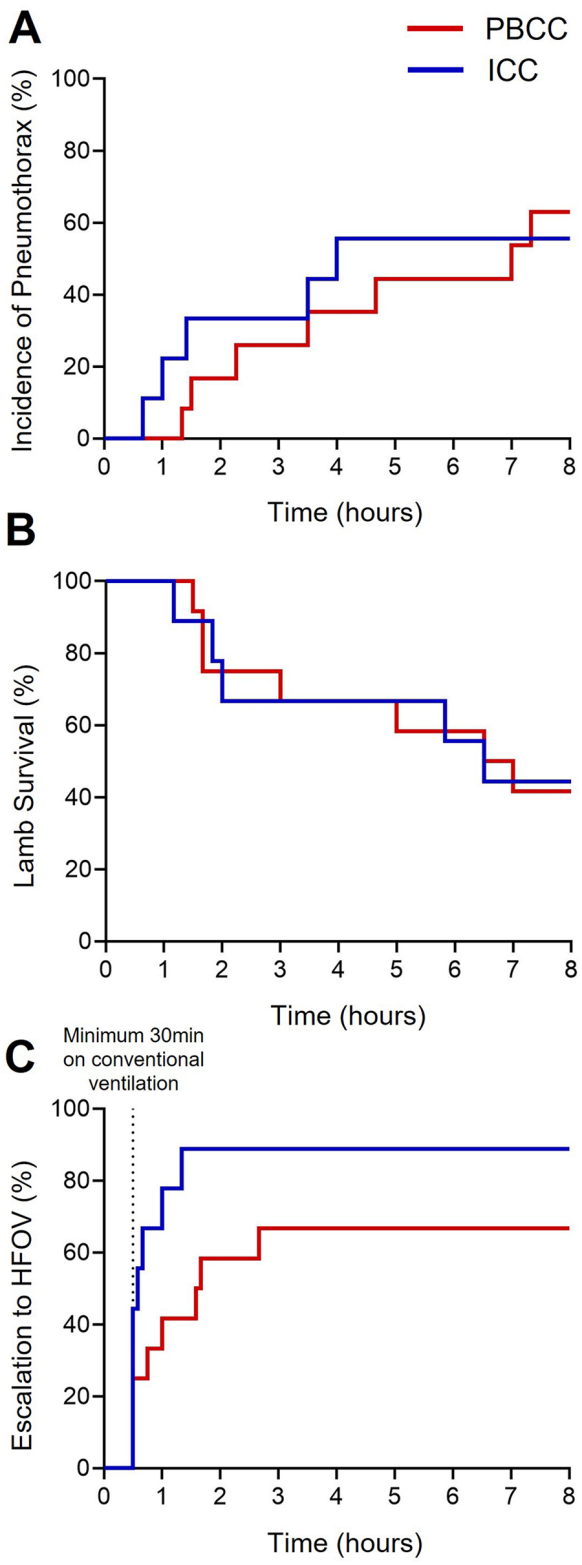
Survival curves of the ICC and PBCC DH lambs during the 8-h ventilation period. Time-related incidences of **(A)** pneumothoraxes (%), **(B)** survival (%), and **(C)** escalation to high-frequency oscillatory ventilation (HFOV, %) measured over the 8-h ventilation period in lambs with a diaphragmatic hernia (DH) receiving immediate cord clamping (ICC, blue) or physiologically based cord clamping (PBCC, red). Log-rank test for all curves (*p ≤ 0.05).

The proportion of DH lambs that developed a treatment-resistant pneumothorax increased over the 8-h ventilation period although this incidence was not different between the groups (Figure 1A; ICC = 55% and PBCC = 58%, *p* = 0.92). As a treatment-resistant pneumothorax was a humane end-point of the study, survival diminished over the 8-h ventilation period (Figure 1B; *p* = 0.97). At the end of the 8-h ventilation period, 88% of the ICC DH lambs required HFOV compared to 66% of the PBCC DH lambs although this difference failed to reach statistical significance (Figure 1C; *p* = 0.08).

### 3.5 Cardiopulmonary physiology throughout the 8-h ventilation period

#### 3.5.1 Pulmonary blood flow

**Figure 2:**
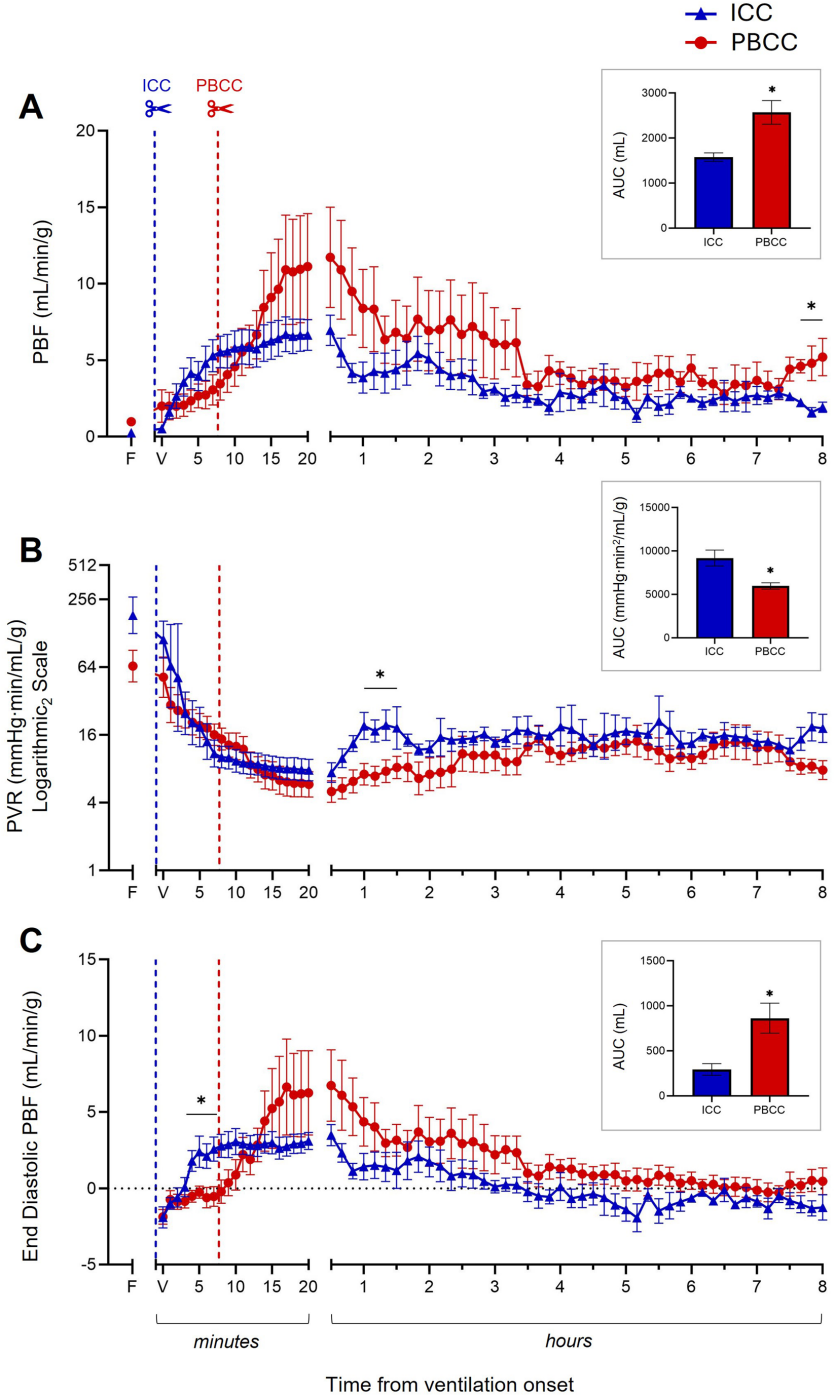
Cardiopulmonary physiology during the 8-h ventilation period. Pulmonary blood flow (PBF, **A**), pulmonary vascular resistance (PVR, **B**, log scale), and end-diastolic PBF (**C**) measured over the 8-h ventilation period in lambs with a diaphragmatic hernia (DH) receiving immediate cord clamping (ICC, blue triangles) or physiologically based cord clamping (PBCC, red circles). Timing of umbilical cord clamping relative to ventilation onset shown by blue (ICC; cord clamped prior to ventilation onset) or red (PBCC; cord clamped when a tidal volume of 4 ml/kg was achieved or at a maximum of 10 min) dotted line. Data are expressed as mean ± SEM. In the ICC DH lambs, *n* = 9 at ventilation onset and *n* = 4 at 8 h, whereas in the PBCC DH lambs, *n* = 12 at ventilation onset and *n* = 4 at 8 h. Mixed-effects analysis with Holm–Sidak multiple comparisons, *p < 0.05 for the effect of group (ICC vs. PBCC). Area under the curve with *t*-test shown in bar graph where *p < 0.05 for ICC vs. PBCC over the 8 h. F = fetal measurement before ventilation onset, V = ventilation onset.

PBF rapidly increased following both ICC and PBCC in the DH lambs (Figure 2A). PBF tended to be greater in the PBCC DH lambs compared to the ICC DH lambs at each timepoint from ∼15 min after ventilation onset until the end of the 8-h ventilation period (Figure 2A). As a result, when measured over the entire 8-h ventilation period (AUC), cumulative pulmonary blood flow was significantly greater in the PBCC DH lambs compared to the ICC DH lambs (2,570 ± 263 vs. 1,578 ± 94 ml, *p* = 0.003; Figure 2A insert). Furthermore, PBF was significantly greater at 8 h after ventilation onset in the PBCC DH lambs compared to the ICC DH lambs (5.2 ± 1.2 vs. 1.9 ± 0.3 ml/min/g, *p* = 0.04; Figure 2A).

#### 3.5.2 Pulmonary vascular resistance

PVR decreased rapidly after ventilation onset in both the ICC and PBCC DH lambs (Figure 2B). PVR tended to be lower in the PBCC DH lambs compared to the ICC DH lambs at each timepoint from ∼15 min after ventilation onset until the end of the 8-h ventilation period (Figure 2B). As a result, when measured over the entire 8-h ventilation period, overall PVR was significantly lower in the PBCC DH lambs compared to the ICC DH lambs (AUC: 5,968 ± 364 vs. 9,188 ± 916 mmHg·min^2^/mL/g, *p* = 0.0002; Figure 2B insert). PVR was significantly lower between 60 and 90 min after ventilation onset and was 2.6-fold lower in the PBCC DH lambs compared to the ICC DH lambs (8 ± 1.7 vs. 21 ± 6 mmHg·min/mL/g, *p* = 0.09; Figure 2B) at 8 h, although this difference was not statistically significant.

#### 3.5.3 End-diastolic pulmonary blood flow

End-diastolic PBF increased following both ICC and PBCC in the DH lambs (Figure 2C). This increase was significantly higher in the ICC DH lambs at 4–7 min post ventilation onset. End-diastolic PBF was then higher in the PBCC DH lambs at 14 min, although this did not reach significance, and remained higher for the duration of the 8-h ventilation. End-diastolic PBF became retrograde (assigned a negative value) in the ICC DH lambs at ∼3.5 h after ventilation onset compared to a positive end-diastolic PBF in the PBCC DH lambs. As a result, a significantly larger volume of blood flowed from left to right across the ductus arteriosus (DA; assigned a positive value) throughout the 8 h in the PBCC DH lambs compared to the ICC DH lambs (AUC: 863 ± 167 vs. 294 ± 65 ml, *p* = 0.008; Figure 2C insert). Although end-diastolic PBF was not significantly different at 8 h after ventilation onset, retrograde flow was present in 75% of the ICC DH lambs and in only 25% of the PBCC DH lambs (−1.3 ± 0.8 vs. 0.5 ± 0.9 ml/min/g; *p* = 0.26).

#### 3.5.4 Systemic and pulmonary blood pressure and cerebral oxygenation

**Figure 3:**
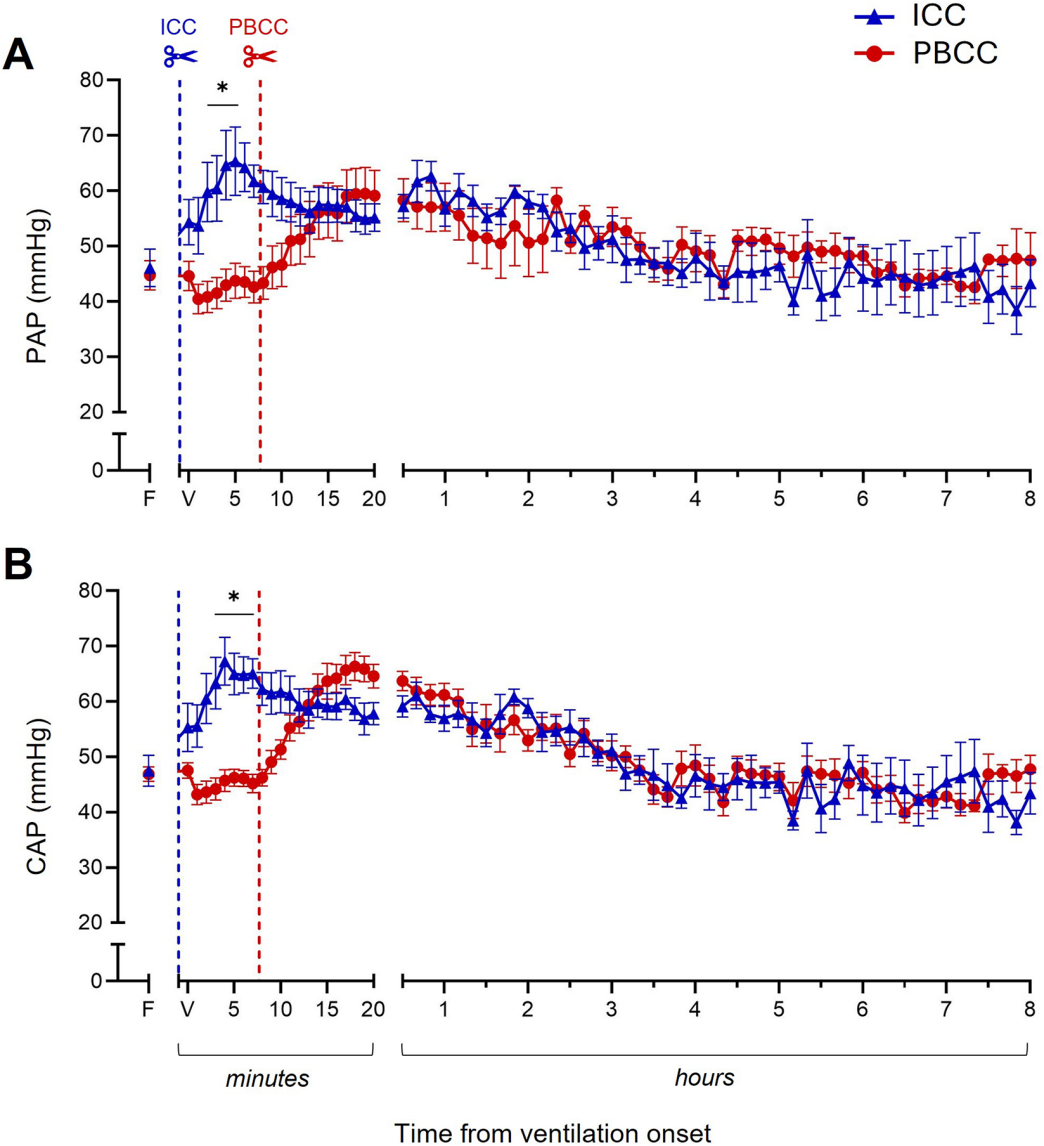
Pulmonary and systemic pressures during the 8-h neonatal ventilation. Pulmonary artery pressure (PAP, **A**) and carotid artery pressure (CAP, **B**) measured over the 8-h ventilation period in lambs with a diaphragmatic hernia (DH) receiving immediate cord clamping (ICC, blue triangles) or physiologically based cord clamping (PBCC, red circles). Timing of umbilical cord clamping relative to ventilation onset shown by blue (ICC; cord clamped prior to ventilation onset) or red (PBCC; cord clamped when a tidal volume of 4 ml/kg was achieved or at a maximum of 10 min) dotted line. Data are expressed as mean ± SEM. In the ICC DH lambs, *n* = 9 at ventilation onset and *n* = 4 at 8 h, whereas in the PBCC DH lambs, *n* = 12 at ventilation onset and *n* = 4 at 8 h. Mixed-effects analysis with Holm–Sidak multiple comparisons, *p < 0.05 for the effect of group (ICC vs. PBCC). F = fetal measurement before ventilation onset, V = ventilation onset.

**Figure 4:**
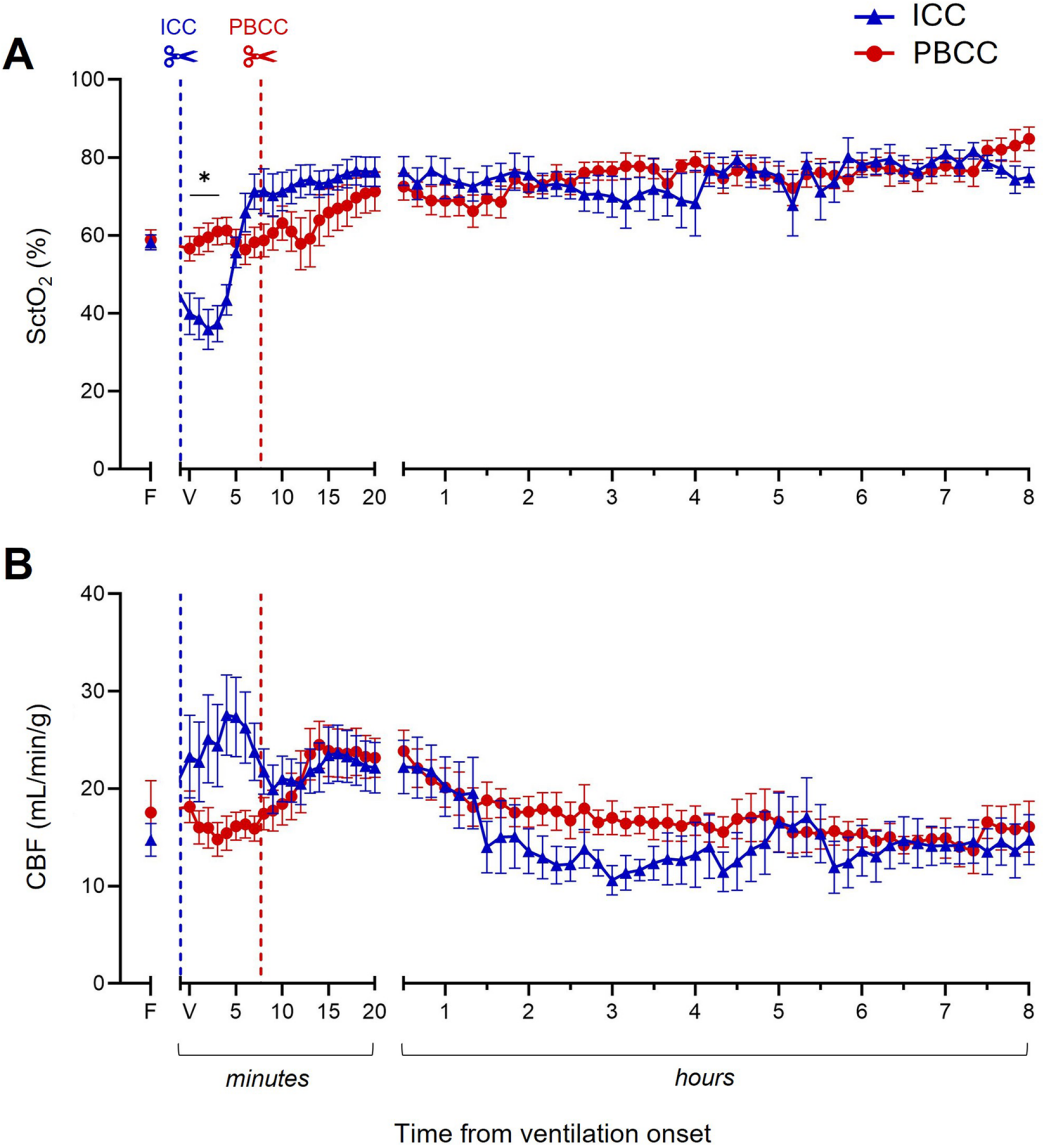
Cerebral oxygenation and carotid blood flow (CBF) during the 8-h neonatal ventilation. Cerebral tissue oxygen saturation (SctO_2_, **A**) and CBF **(B)** measured over the 8-h ventilation period in lambs with a diaphragmatic hernia (DH) receiving immediate cord clamping (ICC, blue triangles) or physiologically based cord clamping (PBCC, red circles). Timing of umbilical cord clamping relative to ventilation onset shown by blue (ICC; cord clamped prior to ventilation onset) or red (PBCC; cord clamped when a tidal volume of 4 ml/kg was achieved or at a maximum of 10 min) dotted line. Data are expressed as mean ± SEM. In the ICC DH lambs, *n* = 9 at ventilation onset and *n* = 4 at 8 h, whereas in the PBCC DH lambs, *n* = 12 at ventilation onset and *n* = 4 at 8 h. Mixed-effects analysis with Holm–Sidak multiple comparisons, *p < 0.05 for the effect of group (ICC vs. PBCC). Area under the curve with *t*-test shown in bar graph where *p < 0.05 for ICC vs. PBCC over the 8 h. F = fetal measurement before ventilation onset, V = ventilation onset.

The lambs that received PBCC avoided the rapid increase in pulmonary artery pressure (PAP; Figure 3A) and carotid artery pressure (CAP; Figure 3B) observed in the ICC DH lambs following umbilical cord clamping. Following ventilation onset, while PAP and CAP did eventually increase in the PBCC DH lambs, this was only after umbilical cord clamping and the increase was considerably slower. PAP and CAP stabilized in both the ICC and PBCC DH lambs by ∼30 min and were similar in both groups throughout the remaining 8-h ventilation period (Figures 3A,B). Similarly, SctO_2_ (Figure 4A) levels were markedly reduced following umbilical cord clamping in the ICC DH lambs, taking >5 min to return to preclamping levels, despite the lambs being ventilated with 73% ± 8% oxygen. However, this decrease was completely avoided by PBCC. Following the first 20 min of ventilation, SctO_2_ levels were similar in the ICC and PBCC DH lambs for the remaining 8 h of ventilation (Figure 4A). Carotid blood flow (CBF; Figure 4B) levels were rapidly increased by umbilical cord clamping in the ICC DH lambs and took almost 10 min to return to similar levels as those in the PBCC DH lambs. Following these initial fluctuations, CBF appeared to be more stable throughout the 8-h ventilation period in the PBCC DH lambs, resulting in a higher cumulative carotid blood flow over the entire 8-h ventilation period in the PBCC DH lambs compared to the ICC DH lambs (AUC: 8,146 ± 244 vs. 7,201 ± 291 ml, *p* = 0.01; Figure 4B).

### 3.6 Arterial blood gas status

**Figure 5:**
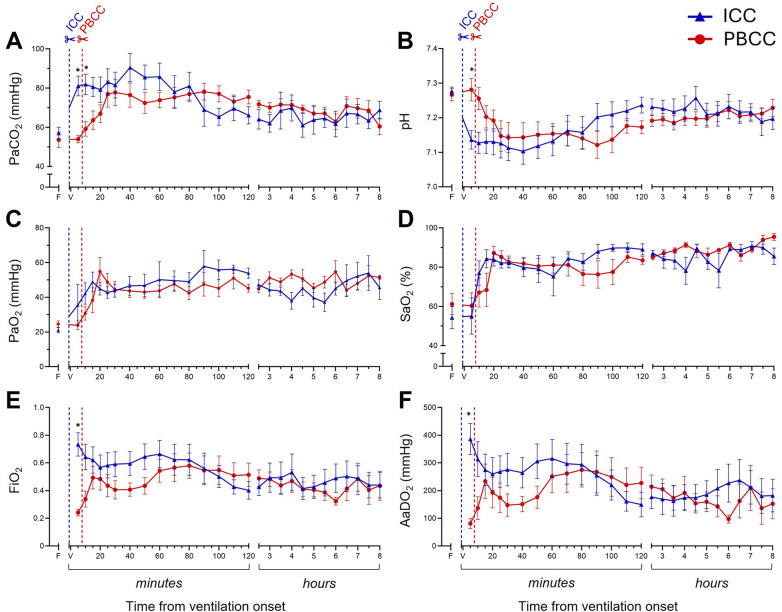
Blood gas status and gas exchange efficiency during the 8-h neonatal ventilation. Partial pressure of carbon dioxide (PaCO_2_, **A**), pH **(B)**, partial pressure of oxygen (PaO_2_, **C**), arterial oxygen saturation (SaO_2_, **D**), fraction of inspired oxygen (FiO_2_, **E**), and alveolar-arterial difference in oxygen (AaDO_2_, **F**) measured over the 8-h ventilation period in lambs with a diaphragmatic hernia (DH) receiving immediate cord clamping (ICC, blue triangles) or physiologically based cord clamping (PBCC, red circles). Timing of umbilical cord clamping relative to ventilation onset shown by blue (ICC; cord clamped prior to ventilation onset) or red (PBCC; cord clamped when a tidal volume of 4 ml/kg was achieved or at a maximum of 10 min) dotted line. Data are expressed as mean ± SEM. In the ICC DH lambs, *n* = 9 at ventilation onset and *n* = 4 at 8 h, whereas in the PBCC DH lambs, *n* = 12 at ventilation onset and *n* = 4 at 8 h. Mixed-effects analysis with Holm–Sidak multiple comparisons, *p < 0.05 for the effect of group (ICC vs. PBCC). F = fetal measurement before ventilation onset, V = ventilation onset.

While PaCO_2_ (Figure 5A) levels increased and pH levels decreased (Figure 5B) in both groups following ventilation onset, initially, the increase in PaCO_2_ and decrease in pH was greater in the ICC DH lambs compared to the PBCC DH lambs. After ∼20 min, PaCO_2_ and pH levels remained similar for the remainder of the 8-h ventilation. PaO_2_ (Figure 5C) and SaO_2_ (Figure 5D) levels increased in both groups following ventilation onset and were similar between the groups for the entire ventilation period. Initially, the FiO_2_ (Figure 5E) delivered to the ICC DH lambs was significantly greater after ventilation onset, but this difference gradually diminished following cord clamping in the PBCC DH lambs due to a gradual increase in their FiO_2_ requirement. After ∼20 min, FiO_2_ levels were similar between the groups for the remaining 8-h ventilation period. Similarly, the AaDO_2_ (Figure 5F) was significantly greater in ICC DH lambs immediately following ventilation onset, largely because the PBCC DH lambs had an intact umbilical cord and did not require supplementary O_2_. Following cord clamping in the PBCC DH lambs, AaDO_2_ decreased similarly to the ICC DH lambs and gas exchange efficiency remained similar between the groups for the 8-h ventilation period.

## 4 Discussion

This study demonstrated that lung aeration before umbilical cord clamping in DH lambs promoted a greater increase in PBF and enhanced the reduction in PVR after birth compared to DH lambs exposed to ICC. This resulted in a predominantly left-to-right flow across the DA in the PBCC DH lambs, which is indicative of a low PVR, compared to the reoccurrence of a right-to-left flow in the ICC DH lambs. Interestingly, these effects were sustained for at least 8 h after ventilation onset, expanding upon our previous studies. This study also confirmed that PBCC in the DH lambs avoided the transient severe hypoxia associated with ICC and the subsequent variations in pulmonary and systemic pressures immediately after birth.

The physiological benefits of PBCC in stabilizing the cardiopulmonary transition at birth are now well established (23, 24), and this study demonstrated that these benefits are sustained in lambs with a DH. In particular, we have previously shown that PBCC results in a higher PBF and lower PVR that persists for at least the first 2 h after birth (18), and have now shown that this benefit is sustained for up to 8 h after birth. While it is often considered that the gradual development of pulmonary hypertension in CDH infants after birth results from abnormal development of the pulmonary vascular bed, it is possible that ICC increases the risk by placing infants on a path toward the development of PPHN. While PBCC may not prevent PPHN in CDH infants, it may mitigate some of the risk.

PPHN is characterized by a sustained elevation of PVR that results in a right-to-left shunting of blood across the DA, from the pulmonary circulation to the systemic circulation (25). In this study, we hypothesized that PBCC would result in a larger reduction of PVR in DH lambs, compared with DH lambs receiving ICC, and that PVR would remain lower for the 8-h ventilation period. While ventilation reduced PVR in both groups, we found that PBCC reduced PVR to a greater degree, and this greater reduction persisted for the duration of the 8-h ventilation period. This greater reduction in PVR was reflected by a positive end-diastolic PBF in the PBCC DH lambs, which is due to a left-to-right shunting of blood across the DA (systemic-to-pulmonary flow) and is a normal characteristic of the cardiopulmonary transition at birth. In contrast, while left-to-right shunting was present in 70% of the PBCC lambs at 3.5 h after ventilation onset, it was absent in 80% of ICC lambs. At 8 h after ventilation onset, right-to-left shunting was present in 75% of the ICC DH lambs. Although end-diastolic PBF did approach zero in the PBCC DH lambs towards the end of the 8-h ventilation period, this could also reflect a gradual closure of the DA.

A study using the CDH Study Group registry observed evidence of pulmonary hypertension, via echocardiography, in 86.5% (1,472 patients) of CDH infants, with 67% of these echocardiograms being performed on the day of birth (11). Clinically, early echocardiography assessment of pulmonary hypertension is logistically challenging and is therefore recommended within the first 24–48 h after birth (13, 14, 26). With the primary outcome of pulmonary hypertension in the first 24 h after birth, the PinC trial (27) assesses both clinical and echocardiographic parameters to investigate whether PBCC can reduce the risk of pulmonary hypertension in CDH infants. Our findings support the concept underpinning the PinC trial, demonstrating that PBCC leads to an overall reduction in PVR during the first 8 h after birth. However, it is important to recognize that the patency of the DA can dramatically change over this time, and when patent, the DA has a major influence on the pressures and flow in the pulmonary arteries. Indeed, when the DA is open, pulmonary arterial pressures must remain similar to systemic arterial pressures as the DA is a very effective low-resistance shunt between the two circulations. As a result, this high pulmonary arterial pressure is not an indicator of PPHN as these high pressures can co-exist with a low PVR due to the high rates of left-to-right shunting across the DA. A high PVR can also co-exist with similarly high pulmonary arterial pressures, due to high rates of right-to-left shunting across the DA, just as occurs in the fetus; in this situation, the DA is commonly regarded as a “pressure relief valve” for the pulmonary circulation.

As reported in the DH lambs, when the umbilical cord is clamped before lung aeration, the sudden removal of the placental circulation causes transient severe hypoxia as the hypoplastic lungs are slow to aerate and take on the role of gas exchange (18). While hypoxia-induced vasoconstriction could have contributed to the higher PVR in the ICC DH lambs, we would expect PVR to be similar between the groups once the preductal saturations stabilized and were maintained within clinical targets of 80%–95% (14). However, while oxygen saturations were similar between groups from ∼30 min after ventilation onset, PVR increased steadily in the ICC DH lambs compared to the PBCC DH lambs. These increases in PVR were closely reflected by decreases in PBF and end-diastolic PBF (18), with the latter indicating the direction of the ductal shunt. The gradual shift to a negative end-diastolic PBF in the ICC DH lambs indicates that the higher PVR reversed the pressure gradient and therefore the blood flow across the DA, leading to a predominantly right-to-left flow as occurs prenatally. It is unclear what mechanisms may be driving this later increase in PVR in the ICC DH lambs, although it could be related to the arterial pressure surge experienced by both the pulmonary and systemic circulations (28).

An array of pulmonary vasodilator therapies exists for the treatment and management of CDH-related pulmonary hypertension, including inhaled nitric oxide, sildenafil, and milrinone (14), although the altered pulmonary vasoreactivity has contributed to the varied effectiveness of these treatments (8). Recent studies assessing the vasoreactivity of CDH vasculature offer promising suggestions for targeted vasodilation therapeutics (29).

The ventilation protocol used in this study is consistent with our previous studies on DH lambs (17, 18) and clearly show that DH lambs require significantly greater PIPs than control lambs to achieve target tidal volumes due to their severely hypoplastic and incompliant lungs (17). In our previous study (17), the target tidal volume of 4 ml/kg was only achieved in two of seven DH lambs when using a PIP limit of 35 cmH_2_O along with the same sustained inflation protocol. While this high PIP limit may have contributed to a greater incidence of pneumothoraces in our DH lambs, the majority of the pneumothoraces occurred in the latter hours when the lambs were receiving HFOV. While this ventilation protocol differs from current clinical recommendations (14), which guide neonatal management in the PinC trial (27), our study was designed to address a specific scientific question. That is, do the benefits associated with resuscitation of DH lambs with an intact cord persist for 8 h after birth (17, 18). As such, this study was designed to replicate ventilation strategies we have used previously, not those used clinically.

As lung aeration is the primary trigger for the reduction in PVR that occurs at birth, the ventilation protocol used (clinically or experimentally) must at least partially aerate the lung for this decrease to occur. As the ventilation pressures we required to aerate the severely hypoplastic lungs of our DH lambs exceed clinically recommended levels, it is possible that the benefits we observed experimentally may not be observed clinically simply because the ventilation protocol is unable to sufficiently aerate the lung and decrease PVR. However, it is important to recognize that a global (whole lung) reduction in PVR only requires partial aeration of the lung (30). While this can lead to a large ventilation/perfusion mismatch in unaerated lung regions, the resulting increase in pulmonary venous return is vital for maintaining left ventricular output (30). This is relevant for CDH infants in whom non-uniform lung aeration and ventilation/perfusion mismatches are common, particularly as the degree of lung aeration achieved using the recommended ventilation pressures is likely to be highly variable between infants. Nevertheless, a recent prospective, single center study in CDH infants has shown that PIPs of 20–25 cmH_2_O (as per clinical recommendations) achieved expired tidal volumes of 1.33 ml/kg at 1 min, increasing to 4.27 ml/kg at 10 min after birth (31). While this tidal volume (4 ml/kg) would be expected to decrease PVR (as seen in our study), the time taken to achieve this volume and decrease PVR highlights the need for prolonged PBCC times to achieve the full benefit. While infants in the prospective study (31) underwent immediate umbilical cord clamping, if the maximum of 10 min of PBCC was applied [as in the PinC trial (27)], these infants would have achieved adequate lung aeration and potentially the benefit of reduced PVR as indicated by our study.

From 30 min after ventilation onset, HFOV was used as a “rescue” therapy if the target ranges for permissive hypercapnia (60–80 mmHg) could not be maintained with conventional mechanical ventilation. Although the severity of lung hypoplasia (measured by the lung-to-body weight ratio) was identical in both the ICC and PBCC DH lambs, fewer PBCC DH lambs (66%) required HFOV compared to the ICC DH lambs (88%). The degree of lung hypoplasia experienced by the lambs in this study is regarded as very severe [∼60% smaller than the control lambs in a previous study (17)] and is consistent with our previous studies of DH lambs (17, 18, 32, 33). In humans, term infants with hypoplastic lungs, defined as an LBWR ≤0.012 for infants ≥28 weeks of gestation, have a lung size equivalent to a healthy fetus of 20–22 weeks of gestation (34). Thus, it is not surprising that our rates of pneumothorax were high, despite the switch to HFOV. Indeed, 28% of the DH lambs developed a treatment-resistant pneumothorax within the first 2 h of ventilation, with an additional 33% of the DH lambs developing a pneumothorax between 2 and 8 h. As a result, animal numbers were low during the later hours of ventilation, but nevertheless we were still able to detect a difference in PBF between the groups at 8 h. However, it is possible that the sickest lambs developed a treatment-resistant pneumothorax, leading to an underestimation of pulmonary hypertension in the DH lambs at 8 h after birth.

In this study, we ventilated the PBCC DH lambs for 7.8 ± 0.8 min before clamping the cord and found, as we have shown previously (18), that PBCC appears to smoothen the cardiopulmonary transition to neonatal life. Specifically, it resulted in lower and much slower increases in arterial pressure and cerebral blood flow and it also avoided the transient hypoxia, hypercapnia, and acidemia associated with ICC. In addition, the findings from this study indicate a reduced PVR and increased PBF that is sustained for at least 8 h after birth. A recent study has suggested that the ventilation of preterm lambs for longer periods (5 min) during delayed cord clamping (i.e., during PBCC) reduces systemic arterial flow compared to shorter period of ventilation with an intact umbilical cord (40 or 120 s) (35). However, we could not find any evidence of an adverse impact of ventilation during PBCC in our study. We did observe a small non-significant decrease in CBF following cord clamping, which is associated with a small decrease in systemic arterial pressure. This finding was caused by the rapid decrease in PVR associated with lung aeration, which redirects right ventricular output toward the lungs and thereby reduces its contribution to systemic arterial flow (15). Nevertheless, as this small decrease in CBF coincided with a small increase in oxygenation (SctO_2_), cerebral oxygen delivery remained unchanged. Furthermore, in response to an article (35), a Letter to the Editor (36) has questioned the validity of their analysis and demonstrated that if the data are plotted in “real time,” systemic arterial flows remain higher and are not reduced in the longer ventilation group.

In summary, we found that the higher PBF and lower PVR following PBCC were sustained for at least 8 h after ventilation onset. While pulmonary hypertension in CDH is most commonly explained by lung hypoplasia and a compromised pulmonary vasculature, all the DH lambs in this study had very severe lung hypoplasia and yet PBCC appeared to mitigate some of the risks associated with PPHN. To further improve outcomes for CDH infants, studies assessing the vasoreactivity of CDH vasculature in response to ventilation and cord clamping could explain the mechanisms driving the PBF and PVR differences observed in this study.

## Data Availability

The raw data supporting the conclusions of this article will be made available by the authors, without undue reservation.
